# Cognitive Decline in Chronic Inflammatory Conditions: Exploring Links Between Systemic Inflammation and Neurodegeneration

**DOI:** 10.7759/cureus.88397

**Published:** 2025-07-21

**Authors:** Julio Joel Jaramillo Ramos, Néstor Manuel Galindo Pupo, Diego Mena, Ricardo Perez Solis, Julian Eduardo Bedoya Jaramillo, Manrique Vega Solano

**Affiliations:** 1 Internal Medicine, Hospital Santo Tomás, Panama City, PAN; 2 Internal Medicine, Universidad Cooperativa de Colombia, Florencia, COL; 3 General Medicine, Pontificia Universidad Católica del Ecuador, Quito, ECU; 4 Material Sciences, Instituto Tecnológico Superior de Atlixco - TecNM, Atlixco, MEX; 5 Internal Medicine, Universidad San Sebastián, Calama, CHL; 6 Emergency Medicine, Vegas Medical Group, San José, CRI

**Keywords:** autoimmune diseases, chronic inflammation, cognitive decline, neurodegeneration, systemic inflammatory markers

## Abstract

Chronic inflammatory diseases (CIDs), such as rheumatoid arthritis (RA), obesity, type 2 diabetes mellitus (T2DM), systemic lupus erythematosus (SLE), fibromyalgia (FM), and chronic infection, are risk factors for neurodegenerative diseases, such as Alzheimer's disease (AD) and Parkinson's disease (PD). This review synthesizes evidence from longitudinal cohort studies and clinical trials, highlighting the contrasting evidence to explain the complex association between systemic inflammation and neurodegeneration. These important mechanisms are disruption of the blood-brain barrier, microglial activation, cytokine-related neurotoxicity (e.g., IL-6, TNF-alpha), lysosomal maladaptation, and metabolic imbalance (e.g., insulin resistance, hyperglycemia). Disease-specific correlations present study outcomes that are paradoxical, including that RA has genetic protection against PD but an increased risk of AD, whereas obesity and T2D associations are the same across studies, persistent associations of these terms with worsened cognitive decline. The risk of dementia is worsened by autoimmune diseases, such as SLE and FM, involving neuroinflammation caused by autoantibodies and central sensitization in the latter, respectively. The new markers, such as glial fibrillary acidic protein (GFAP) (neuroinflammation) and neurofilament light (NfL) (axonal injury), have prospects in early detection, but there is a need for validation in future studies. Therapeutic approaches emphasize the need for immunomodulation (e.g., disease-modifying antirheumatic drugs {DMARDs} in RA), glycemic control in T2DM, and lifestyle interventions, with a lack of targeting the non-amyloid pathways. New studies should emphasize longitudinal studies, multiome-based methods, and clinical trials in an attempt to create precision treatment. Considering the inflammatory risk stratification in the prevention of neurodegeneration, this review sheds light on the possibility of early preventative action that may offset the progressive cognitive decline in especially vulnerable groups.

## Introduction and background

Chronic inflammatory diseases, including rheumatoid arthritis, inflammatory bowel disease, and systemic lupus erythematosus, are defined by persistent systemic inflammation that is not restricted to local tissue destruction [1]. Indications of the possibility of certain neurodegenerative processes being driven by chronic exposure to inflammatory mediators, which cause cognitive decline, have emerged [2]. Both Alzheimer's disease and Parkinson's disease are neurodegenerative processes that share comparable inflammatory pathways with combined periphery diseases, which brings the possibility that the rise of systemic inflammation may be a part of age-related brain dysfunction in addition to deterioration [3].

These neurodegenerative and systemic inflammatory processes are linked and are also related to the complex of mechanisms, damage to the blood-brain barrier (BBB), microglia activation, and secretion of such pro-inflammatory mediators, including tumor necrosis factor-alpha (TNF-alpha), interleukin-6 (IL-6), and C-reactive protein (CRP) [4,5]. The constant rise of these inflammatory markers has been attributed to synaptic dysfunction, neuronal loss, and an excess of pathological proteins, namely amyloid-beta and -tau. Cogent knowledge of these mechanisms is significant in determining possible treatment alternatives whereby the degradation of cognitive decline can be reduced among patients with chronic inflammatory diseases [6,7].

This review reflects on the existing evidence of an interconnection between systemic inflammation and neurodegeneration, with a focus on the paths of chronic immune activation that contribute to the onset of cognitive impairment [8]. The importance of this review is that it could help to fill the gaps between immunology and neuroscience and provide information about common pathological mechanisms [9]. Moreover, the process of identifying populations at risk may result in early interventions, including anti-inflammatory treatment and lifestyle changes, to maintain cognitive ability [10].

This review is focused on understanding that neurodegenerative diseases might be associated with chronic inflammatory illness as a modifiable risk factor. This review synthesizes the existing literature on chronic inflammation as a risk factor for cognitive impairment. It also aimed to give a better understanding of how systemic inflammation leads to cognitive decline and identify the most critical molecules involved in this process and the clinical implications of prevention and treatment options. This review may open the door to new avenues to preserve brain health in patients with chronic inflammatory diseases.

## Review

Methodology

The literature review was conducted using the Population, Intervention, Comparison and Outcomes (PICO) framework, with the following keywords used for the "Population" component: rheumatoid arthritis (RA), Parkinson’s disease (PD), Alzheimer’s disease (AD), chronic kidney disease (CKD), obesity, fibromyalgia (FM), multiple sclerosis (MS), and chronic obstructive pulmonary disease (COPD). Common mechanisms linking inflammation to neurodegeneration (exposure) include IL-6, TNF-α, C-reactive protein (CRP), interleukins (IL-1β, IL-12), amyloid-β, tau, neurofilament light chain (NfL), and glial fibrillary acidic protein (GFAP). The Comparison involves different diseases, and the outcomes assessed are memory, cognitive functions, dementia risk, and cognitive decline. The literature was searched on PubMed and Google Scholar Journals using the PICO framework. The data were extracted using the Cochrane characteristics of included studies guidelines to extract topic-relevant data. Findings of selected studies were combined using the inductive data-driven synthesis approach with emphasis on the interpretation of the mechanisms between the chronic inflammatory diseases and the neurodegeneration. The results were presented using each study's p-value at a 95% confidence interval to indicate statistical significance between the neurodegenerative disease and chronic inflammatory conditions.

Results and discussion

Study Characteristic

The 22 studies included in the review employ diverse methodologies to investigate the link between chronic inflammatory conditions and neurodegeneration. Chen et al. and Li et al. both utilized randomization studies focusing on rheumatoid arthritis (RA), highlighting systemic inflammation and lysosomal dysfunction as key mechanisms, with RA showing a protective genetic correlation against Parkinson’s disease (PD) [11,12]. In contrast, Kodishala et al. conducted a population-based cohort study of 886 RA patients, finding no significant association between RA disease activity and dementia risk, possibly due to limited racial diversity and retrospective design [13].

Longitudinal designs were common in obesity-related studies. Whitmer et al. reported a 74% increased dementia risk in obese individuals (HR: 1.74, 95% CI: 1.34-2.26), while Dahl et al. found higher late-life BMI associated with lower dementia risk, suggesting reverse causality or preclinical weight loss bias [14,15]. For metabolic disorders, Marden et al. and Tuligenga et al. emphasized hyperglycemia and insulin resistance as drivers of cognitive decline, with poor glycemic control exacerbating memory decline (β=-0.04/decade) [16,17].

Autoimmune conditions like systemic lupus erythematosus (SLE) were examined in nationwide cohort studies. Lin et al. reported a 2.14 times higher dementia risk in SLE patients, attributed to cerebrovascular and autoimmune pathways, while Kim et al. found higher PD risk, implicating neuroinflammation and dopaminergic dysfunction [18,19]. Fibromyalgia (FM) studies, such as Tzeng et al., identified increased dementia risk, linking central sensitization and pro-inflammatory cytokines to neurodegeneration [20]. Viral infections like herpes simplex virus (HSV) were explored by Letenneur et al., where IgM+ status (reactivation marker) correlated with higher Alzheimer’s risk, suggesting chronic brain inflammation as a mechanism [21].

Chronic Inflammatory Conditions

The neurodegenerative diseases have been widely researched in relation to chronic inflammatory diseases, including RA, obesity, type 2 diabetes (T2D), SLE, FM, and chronic infections, e.g., herpes simplex virus (HSV). An example is RA, which has a complex association with neurodegeneration, and systemic inflammation and immune deregulation could predispose LOAD, but potentially be protective in PD because of the high activity of lysosomal enzymes [11,12]. On the contrary, obesity and T2D are systematically connected with increased risk of dementia due to such processes as adipocytokine-mediated inflammation, insulin resistance, and microvascular damage [14,16]. SLE has close connections with dementia and PD, as it is facilitated by autoantibody-related neuroinflammation and cerebral vascular injury [18,19]. With PD, fibromyalgia is a common comorbidity that has dopaminergic dysfunction and central sensitization pathways, which leads to cognitive decline [20,22]. The role of chronic infections in amyloid aggregation and hippocampal damage is under debate, with HSV as a possible cause but not cytomegalovirus (CMV) and Epstein-Barr virus (EBV) [21,23].

Mechanisms Linking Inflammation to Neurodegeneration

The mechanisms linking chronic inflammation to neurodegeneration are multifaceted and vary across different inflammatory conditions. Systemic inflammation, as seen in RA, contributes to neurodegeneration through immune responses, blood-brain barrier (BBB) dysfunction, and lysosomal dysfunction [11]. In RA, elevated lysosomal enzyme activity may paradoxically protect against Parkinson’s disease (PD) by mitigating protein aggregation, while systemic inflammation exacerbates Alzheimer’s disease (AD) risk [12]. Similarly, obesity and metabolic disorders like T2DM drive neurodegeneration via adipocytokines, insulin resistance, and chronic hyperglycemia, which induce microvascular injury and neuronal damage [14,16]. Mielke et al. highlight neuroinflammation via glial fibrillary acidic protein (GFAP) and axonal degeneration via neurofilament light chain (NfL) as key pathways in T2D-related cognitive decline, with limited involvement of amyloid/tau pathology [24].

Vascular mechanisms are prominent in conditions like atherosclerosis and hypertension, where hypoperfusion, endothelial dysfunction, and shared risk factors (e.g., hypertension, diabetes) link vascular pathology to cognitive impairment [25,26]. According to Dearborn et al., intracranial atherosclerotic disease (ICAD) increases dementia risk by 3.8-fold, likely due to coexisting AD pathophysiology [25]. Autoimmune diseases, such as SLE and multiple sclerosis (MS), involve neuroinflammation via autoantibodies (e.g., anti-NMDA, anti-dopaminergic) and microglial activation, leading to hippocampal atrophy and dopaminergic neuron loss [18,27]. Gray matter atrophy in MS begins with secondary white matter destruction in the initial stages and primary neurodegeneration in the later stages [28]. There are also hints that chronic infections (e.g., HSV) can cause neurodegeneration via viral-mediated hippocampal injuries and amyloid clustering, yet the evidence on CMV/EBV is inconclusive [21,23].

Inflammatory Markers

The markers of inflammation recognized in numerous chronic inflammatory diseases and their relationships with neurodegenerative disorders are showing the intricate relation between systemic inflammation and brain health. The significant indicators are IL-6, IL-1β, TNF-α, IL-12, IL-18, TGF-β, CRP, adipocytokines, antiphospholipid antibodies, and GFAP, among others [11,12,27]. Indeed, in RA, IL-6, IL-1β, and TNF-α are pointed out in the literature as being overexpressed and correlate with systemic inflammation and blood-brain barrier (BBB) impairment, which may predispose to neurodegenerative diseases, such as late-onset Alzheimer’s disease (LOAD) and PD [11,12]. It is also worth noting that RA presents with increased cathepsins (lysosomal enzymes), which could paradoxically benefit against PD via the modulation of lysosomal activity [13]. Conversely, in obesity research, C-reactive protein and adipocytokines are highlighted, and these are associated with the risk of dementia in midlife, indicating metabolic inflammation as a probable motivation [14].

HbA1c in T2D is a surrogate of chronic inflammation due to hyperglycemia [16,17], whereas GFAP and NfL are novel biomarkers of neuroinflammation and axonal injury, respectively, which do not seem to play a major role in amyloid/tau pathology [24]. Likewise, SLE literature involves the antiphospholipid antibodies and TNF-α in the cerebrovascular and dopaminergic impairment, putting dementia and PD at risk [18,19]. In fibromyalgia (FM) studies, IL-8 and monocyte chemoattractant protein-1 (MCP-1) are indicated in hippocampal atrophy and neural noise [20], whereas HSV IgM could induce persistent neuroinflammation, which doubles the risk of Alzheimer's [21]. In turn, CMV and EBV antibodies were not significantly associated with cognitive decline, which highlights the necessity of further studies of viral triggers [23].

Disease-Specific Associations

The analyzed studies indicate a variety of disease-specific interactions between chronic inflammatory processes and neurodegenerative outcomes. As an example, RA exhibits a complex relationship with neurodegeneration: although RA is associated with an increased risk of LOAD due to systemic inflammation and immune dysregulation [11], it shows a negative genetic correlation with Parkinson’s disease (PD), suggesting possible protective effects, potentially mediated by increased lysosomal enzyme activity [12]. On the contrary, SLE consistently relates to increased risks of both dementia and PD [18], which is probably because of the neuroinflammation and vascular harm produced by autoimmune attacks [19].

Cognitive decline is closely linked with metabolic disorders, such as obesity and T2D. Obesity in midlife is associated with a 74% increased risk of dementia [14], and T2D accelerates memory decline, with poor glycemic control worsening this effect [16,17]. However, late-life obesity has also been associated with reduced risk of dementia, which could be due to reverse causality [15]. Fibromyalgia (FM) is associated with an increased risk of dementia by 2.77-fold [20], whereas CKD is related to a greater rate of cognitive decline due to vascular aging [29]. Viral infections, such as HSV, give disparate findings, where IgM seropositivity (reactivated infection) is a risk factor for Alzheimer's, but CMV and EBV latent infection do not give clear results [21,23].

Biomarkers and Diagnostics

Genetic variants, such as single-nucleotide polymorphisms (SNPs) of GWAS and lysosomal enzymes (cathepsin D), are examined in RA and discussed as candidate protective factors against PD [11,12]. On the contrary, the study of SLE incorporates the use of ICD codes since no direct measurement of biomarkers is available, although anti-NMDA receptor antibodies are suspected in hippocampal injury [18]. In the case of metabolic disorders, such as T2D, HbA1c is a crucial glycemic control and cognitive decline biomarker [16,17], whereas plasma GFAP and NfL are neuroinflammation and axonal damage indicators in T2D and obesity [24]. Research on FM is based only on inflammatory cytokines (e.g., IL-8), which have been suggested as possible biomarkers [20]. Viral infections, such as HSV, are evaluated through IgM/IgG serology, whereby IgM is associated with increased risk of Alzheimer's disease [21], and finally, CMV/EBV researchers report that there is no definite association between any biomarker and cognition [23]. The more direct biomarkers (e.g., NfL, GFAP, HbA1c) are used in MS/T2D studies than in SLE or FM. HbA1c and NfL can be used in the clinical setting; however, the use of genetic variants (e.g., RA) or autoantibodies (e.g., SLE) needs to be validated. There are no strong biomarkers of SLE, FM, and bronchiectasis, which underlines the importance of mechanistic investigations [20,27,30].

Therapeutic Strategies

The strategies of therapy, aimed at reducing neurodegeneration in chronic inflammatory diseases, differ with respect to disease-specific pathogenic processes and disease biomarkers. In the case of RA, mobilizing RA as such (e.g., by bringing systemic inflammation under control) might decrease the risk of LOAD, and the investigation of lysosomal pathways (e.g., cathepsin D modulation) may provide protection against PD [11,12]. Conversely, the focus of obesity and T2D techniques is on glycemic control and lifestyle techniques. Marden et al. pointed out that preservation of glycemic control (HbA1c <7%) could reduce cognitive decline [16], whereas Mielke et al. indicated intensive lifestyle measures (diet, exercise) had minor impacts on the biomarkers of neuroinflammation (GFAP/NfL), indicating the necessity of specific anti-inflammatory treatment [24].

In the case of autoimmune diseases, such as SLE, it is suggested to implement steroid-sparing treatments and vascular risk factor management (e.g., control blood pressure) to decrease the risk of dementia [18], and NSAIDs (e.g., ibuprofen) could be neuroprotective in PD through peroxisome proliferator-activated receptor gamma (PPARγ) signaling [19]. In multiple sclerosis (MS), disease-modifying therapies (DMTs), such as interferon-beta and glatiramer acetate, are efficient in the initial phases of the disease but have little effect on the atrophy of the gray matter in progressive MS [28]. In secondary progressive MS, simvastatin, despite being neuroprotective with respect to brain atrophy, did not change the NfL levels, inspiring the demand for biomarkers that are independent of neuroaxonal injury [31].

Fibromyalgia (FM) has no specific treatments for cognitive decline, although symptomatic treatment (e.g., serotonin-norepinephrine reuptake inhibitors {SNRIs}, selective serotonin reuptake inhibitors {SSRIs}) and anti-inflammatory medications are postulated to improve central sensitization [20,22]. In the case of viral causes of Alzheimer’s disease (AD), such as HSV, antiviral treatment and vaccination are suggested to be preventive measures against Alzheimer’s disease, but there are no clinical trials yet [21]. The comparison of cross-conditions indicates that metabolic disorders (T2D, obesity) are associated with the priority of lifestyle and glycemic control, whereas autoimmune diseases (RA, SLE) are characterized by the preference of immunomodulation and the reduction of vascular risks. These are the lack of evidence of disease-modifying in FM and bronchiectasis, and the necessity of biomarker-guided therapies in MS and T2D. Table 1 illustrates the key variables and summarizes the studies that demonstrate the association between systemic inflammation and neurodegeneration.

**Table 1:** Characteristics and findings of reviewed studies. RA: rheumatoid arthritis; LOAD: late-onset Alzheimer’s disease; PD: Parkinson’s disease; SNPs: single nucleotide polymorphisms; DMARDs: disease-modifying anti-rheumatic drugs; ESR: erythrocyte sedimentation rate; RF: rheumatoid factor; anti-CCP: anti-cyclic citrullinated peptide antibodies; ICD-10: International Classification of Diseases, 10th Revision; MoCA: Montreal Cognitive Assessment; MMSE: Mini-Mental State Examination; DASC-21: Dementia Assessment Scale-21; HbA1c: glycated hemoglobin; eGFR: estimated glomerular filtration rate; 3MS: Modified Mini-Mental State Exam; BMI: body mass index; CRP: C-reactive protein; CMV: cytomegalovirus; EBV: Epstein-Barr virus; TNF-α: tumor necrosis factor-alpha; IL-6: interleukin-6; IL-1β: interleukin-1 beta; IL-12: interleukin-12; IL-18: interleukin-18; TGF-β: transforming growth factor-beta; GWA: Genome-Wide Association; PPARγ: peroxisome proliferator-activated receptor gamma; NfL: neurofilament light chain; NfH: neurofilament heavy chain; APOE-ε4: apolipoprotein E epsilon 4; SNRIs: serotonin-norepinephrine reuptake inhibitors; SSRIs: selective serotonin reuptake inhibitors; FLISPAD: Functional Limbic Interface Syndrome with Pain and Depression; HR: hazard ratio; CI: confidence interval; OR: odds ratio; IGF-1: insulin-like growth factor-1; ICAD: Intracranial Atherosclerotic Disease; RF/anti-(CCP): rheumatoid factor/anti-cyclic citrullinated peptide; SNPs: single nucleotide polymorphisms; MCI: mild cognitive impairment; CVD: cardiovascular disease; ICD-9-CM: International Classification of Diseases, Ninth Revision, Clinical Modification

Studies	Study characteristic	Chronic inflammatory conditions	Mechanisms linking inflammation to neurodegeneration	Inflammatory markers	Disease-specific associations	Biomarkers and diagnostics	Therapeutic strategies	Challenges and gaps	Clinical and public health	Future research
Chen et al. (2024) [11]	Randomized controlled trial study.	Rheumatoid arthritis (RA).	Systemic inflammation, immune responses, and the blood-brain barrier. Dysfunction, lysosomal dysfunction.	IL-6, IL-1β, TNF-α, IL-12, IL-18, TGF-β.	RA increases the risk of LOAD; potential protective effect against Parkinson’s disease.	Amyloid-β, tau, lysosomal enzymes (e.g., cathepsin D).	Proactive rheumatoid arthritis (RA) management for LOAD risk reduction.	Generalizability to non-European populations, small PD cohort, and latent pleiotropy.	Monitoring RA patients for late-onset Alzheimer’s disease (LOAD) and Parkinson’s disease (PD).	Larger cohorts, mechanistic studies, and diverse ethnic groups.
Li et al. (2021) [12]	Randomized controlled trial study.	Rheumatoid arthritis (RA).	Lysosomal dysfunction hypothesis (elevated lysosomal enzyme activity in RA may protect against PD). Immune response and inflammation pathways.	Proinflammatory mediators (e.g., IL-1β, IL-6, cathepsins).	Negative genetic correlation between RA and PD (OR: 0.904, 95% CI: 0.866-0.943).	Genetic variants (SNPs) from GWAS.	Exploration of the lysosome pathway and immune modulation as potential therapeutic targets.	Confounding factors (e.g., smoking, BMI). Population stratification. Limited generalizability to non-European populations.	Reduced risk of Parkinson’s disease (PD) in rheumatoid arthritis (RA) patients suggests potential protective mechanisms.	Mechanisms of lysosomal dysfunction in rheumatoid arthritis (RA) and Parkinson’s disease (PD): longitudinal studies on RA patients with PD - non-European population studies.
Kodishala et al. (2023) [13]	Population-based cohort study of 886 RA patients (1980-2014) followed for dementia risk.	Rheumatoid arthritis (RA).	Systemic inflammation increases blood-brain barrier permeability, activates microglia, and causes neuroinflammation.	Erythrocyte sedimentation rate (ESR) (not significantly associated with dementia).	RA disease activity (large joint swelling, rheumatoid nodules) and cardiovascular disease events are linked to higher dementia risk.	ICD-9/ICD-10 codes for dementia; RF/anti-cyclic citrullinated peptide (anti-CCP) antibodies, an antibody (no significant association).	No significant association found with disease-modifying antirheumatic drugs (DMARDs/biologics); potential confounding by indication.	Limited racial diversity, retrospective design, and lack of apolipoprotein E4 (APOE-ε4) status data.	High-risk phenotype: elderly RA patients with active disease, cardiovascular disease (CVD), or mood disorders.	Role of serial inflammatory markers, apolipoprotein E4 (APOE-ε4) status, and dementia subtypes.
Whitmer et al. (2005) [14]	Longitudinal study on midlife obesity (BMI, skinfold thickness) and dementia risk.	Obesity (BMI ≥30) and overweight (BMI 25.0-29.9).	Cardiovascular disease and diabetes pathways. Direct neuronal degradation via adipocytokines and inflammation. Metabolic syndrome and cognitive decline.	C-reactive protein (CRP), adipocytokines.	Obese individuals: 74% increased dementia risk (HR: 1.74, 95% CI: 1.34-2.26). Overweight individuals: 35% increased risk (HR: 1.35, 95% CI: 1.14-1.60). Highest skinfold thickness quintile: 59-72% increased risk.	Body mass index (BMI), subscapular/triceps skinfold thickness.	Potential reduction of dementia risk through obesity treatment.	Limited data on weight cycling, diet, and nutrition. No waist circumference measures. Possible underdiagnosis of dementia in non-clinic attendees.	Midlife obesity is an independent risk factor for dementia, suggesting public health interventions.	Role of adipocytokines and inflammation in brain structure/function. Impact of central obesity (waist circumference). Confirmation in diverse populations.
Dahl et al. (2008) [15]	Prospective population-based study of 605 elderly Finns (65-92 years) over eight years.	CVD and metabolic diseases.	Preclinical weight loss and metabolic alterations potentially linked with neurodegenerative processes.	CVD and diabetes as comorbidities potentially linked to systemic inflammation.	Higher BMI in late life is associated with a lower risk of all-cause dementia, particularly in women more than 70 years of age. Low BMI is associated with increased risk of dementia.	BMI as a continuous variable; dementia diagnosed via DSM-IV criteria and clinical exams.	Not discussed	Homogeneous (White) cohort, small subgroup sizes, potential preclinical weight loss bias.	High BMI in late life may not be a dementia risk factor; low BMI could signal preclinical dementia.	Need for diverse cohorts, better body composition measures (e.g., waist circumference).
Ishikawa et al. (2024) [26]	Sample: 677 patients, mean age 79.2 years. Methods: cross-sectional and longitudinal analysis using MoCA, MMSE, and DASC-21 scores.	Hypertension, diabetes, dyslipidemia, atrial fibrillation, heart failure, stroke.	Lower BP is linked to reduced ADL (frailty/sarcopenia). Higher BP is associated with probable dementia in non-hypertensive patients. Reverse causality: ADL decline may lead to BP reduction.	Not explicitly mentioned.	Probable dementia (MMSE ≤23) is linked to higher SBP in non-hypertensive patients. Lower SBP is associated with DASC-21 ≥31 (reduced ADL) in hypertensive patients.	MoCA for mild cognitive impairment. MMSE for advanced cognitive dysfunction. DASC-21 for ADL assessment.	Antihypertensive medication may slow MCI progression. Caution is needed in BP management for patients with comorbidities (stroke, heart failure).	Single-center study. Limited follow-up data due to COVID-19. Lack of out-of-clinic BP measurements.	Individualized BP targets for elderly patients. Monitoring cognitive function in hypertensive patients.	Further studies on out-of-office BP and cognitive decline. Larger, multicenter studies with longer follow-up.
Dearborn et al. (2017) [25]	Cross-sectional study on intracranial atherosclerotic disease (ICAD) and cognitive impairment (MCI/dementia).	Atherosclerosis (ICAD).	Large-vessel atherosclerosis contributing to neuronal dysfunction via hypoperfusion. Coexistence of vascular pathology and Alzheimer’s disease (AD) pathophysiology.	Not explicitly mentioned (implied systemic inflammation from atherosclerosis).	ACA plaques: 3.81x higher dementia prevalence (95% CI: 1.57-9.23). PCA plaques: 1.43x higher MCI prevalence (95% CI: 1.04-1.98). Stenosis >50%: 1.92x higher dementia risk (95% CI: 1.01-3.65).	High-resolution vessel wall MRI (BBMRI) and MRA for ICAD detection.	Management of vascular risk factors (e.g., hypertension, LDL). Potential ICAD as a biomarker for dementia risk stratification.	Cross-sectional design (cannot infer causality). Small sample size for dementia subgroup (n=83). Limited racial diversity (underpowered for subgroup analyses).	Intracranial atherosclerotic disease (ICAD) may be a modifiable risk factor for dementia, emphasizing vascular health in cognitive decline prevention.	Longitudinal studies to assess ICAD progression and incident dementia. Mechanistic studies on intracranial atherosclerotic disease (ICAD) and Alzheimer’s disease (AD) (e.g., β-amyloid). Exploration of ICAD in diverse populations.
Darsie et al. (2014) [29]	Prospective cohort study of 3,907 older adults (≥65 years) in the Cardiovascular Health Study (1992-1999).	Chronic kidney disease (CKD).	Vascular aging, subclinical ischemia, and shared risk factors (hypertension, diabetes) may mediate the kidney-cognition link.	C-reactive protein (adjusted for in analysis).	Lower eGFR (<60 mL/min/1.73 m²) is associated with faster cognitive decline (0.64 points/year on 3MS).	Cystatin C-based eGFR (eGFR), 3MS exam, and DSST for cognitive function.	None directly studied; emphasis on managing CKD and cardiovascular risk factors.	Unclear causality, limited follow-up, and lack of urinary albumin data.	Chronic kidney disease (CKD) patients are at higher risk for cognitive decline; monitor kidney and brain health jointly.	Mechanisms (e.g., neurovascular coupling), role of albuminuria, interventions targeting chronic kidney disease (CKD).
Yaffe et al. (2010) [32]	Cross-sectional study of 825 adults aged ≥55 years with CKD; evaluated cognitive function across eGFR strata.	Chronic kidney disease (CKD).	Cerebrovascular disease, metabolic dysregulation, anemia, oxidative stress, and altered lipid/homocysteine metabolism.	Not specified.	Advanced CKD (eGFR <30) is linked to cognitive impairment in global cognition, attention, memory, and executive function.	Glomerular filtration rate, cognitive tests (3MS, Trails A/B, Boston Naming, Buschke Test, Category fluency).	Not discussed.	Cross-sectional design limits causal inferences; lack of brain imaging data.	Suggests screening for cognitive impairment in advanced CKD patients.	Longitudinal studies to explore causality, mechanisms, and interventions.
Mielke et al. (2025) [24]	Longitudinal cohort study on blood-based biomarkers (BBMs) and cognitive decline in adults with T2D and obesity.	Type 2 diabetes (T2D), obesity.	Neuroinflammation (via GFAP). Axonal degeneration (via NfL). Limited role of amyloid/tau pathology in this population.	Glial fibrillary acidic protein (GFAP), (neuroinflammation), NfL (axonal damage).	Rising GFAP/NfL levels are linked to cognitive decline (GFAP: OR: 1.25, 95% CI: 1.10-1.42; NfL: OR: 1.10, 95% CI: 1.03-1.18). No association with Aβ42/40 or pTau-181.	Plasma GFAP, NfL, Aβ42/40, and pTau-181.	Intensive lifestyle intervention (ILI) showed no effect on BBMs. Focus on managing neuroinflammation/axonal health.	Cross-sectional blood-brain barrier model measurements lack prognostic value. Limited generalizability (high-risk cohort only). No baseline cognitive data.	Glial fibrillary acidic protein (GFAP)/neurofilament light chain (NfL) may serve as monitoring tools for cognitive risk in T2D/obesity.	Longitudinal tracking of glial fibrillary acidic protein (GFAP)/ neurofilament light chain (NfL) in diverse populations. Mechanistic studies on neuroinflammation in metabolic disorders. Exploration of combo biomarkers (e.g., GFAP+vascular markers).
Marden et al. (2017) [16]	Longitudinal study of 8,888 U.S. adults (50+ years) from the Health and Retirement Study (2006-2012).	Type 2 diabetes (T2D).	Hyperglycemia (chronic high HbA1c) → microvascular injury; insulin dysregulation → neuronal damage.	Glycosylated hemoglobin (HbA1c).	Type 2 diabetes associated with 10% faster memory decline (β=−0.04/decade), each 1-unit HbA1c increase → 0.05 SD memory decline/decade.	HbA1c, composite memory score (word recall + IQCODE) Informant Questionnaire on Cognitive Decline in the Elderly.	Glycemic control for diabetics; potential interventions for pre-diabetic HbA1c levels.	Self-reported diabetes; single HbA1c measurement; no Alzheimer’s/vascular dementia differentiation.	Monitor HbA1c in diabetics and non-diabetics; early glycemic control may mitigate memory decline.	Mechanisms (e.g., insulin pathways), long-term hemoglobin A1c (HbA1c) trends, and APOE-4 interactions.
Tuligenga et al. (2014) [17]	Design: post hoc analysis of the Whitehall II cohort (prospective). Sample: 5,653 adults (median age 54.4 years).	Type 2 diabetes, prediabetes, hypertension, and obesity.	Vascular pathways: microangiopathy, ischemic lesions. Metabolic dysregulation: hyperglycemia-induced oxidative stress, insulin resistance. Shared risk factors: hypertension, dyslipidemia.	Glycemic control (HbA). Noted as a proxy for chronic metabolic stress.	Known diabetes is linked to faster decline in memory, reasoning, and global cognition. No significant decline in prediabetes/newly diagnosed diabetes. Poor glycemic control (HbA) exacerbated decline.	Cognitive tests: 20-word recall (memory), Alice Heim 4-I (reasoning), and phonemic/semantic fluency. Glycemic control: HbA as a biomarker.	Prevention: lifestyle interventions to delay diabetes onset. Management: tight glycemic control (e.g., HbA <7%) may slow decline (mixed evidence).	Generalizability limited to occupational cohort (healthier than general population). Lack of non-white subgroup analysis. No direct inflammatory marker measurement.	Screen middle-aged diabetics for cognitive decline. Emphasize glycemic control and cardiovascular risk management.	Investigate inflammatory biomarkers (e.g., C-reactive protein {CRP}, IL-6) in diabetes-related cognitive decline. Trials on anti-diabetic drugs (e.g., metformin) and cognitive outcomes. Longitudinal studies with neuroimaging to assess vascular vs. neurodegenerative pathways.
Fisher et al. (2008) [28]	Longitudinal study on gray matter (GM) atrophy in multiple sclerosis (MS) patients.	Multiple sclerosis (MS).	GM atrophy driven by focal/diffuse tissue damage, possibly secondary to white matter (WM) pathology in early stages, primary GM pathology dominates in later stages.	T2 lesion volume (T2LV), T1 hypointense lesion volume (T1LV), gadolinium-enhancing lesions, magnetization transfer ratio (MTR) of normal-appearing brain tissue (NABT), and lesions.	GM atrophy rates increase with disease stage (clinically isolated syndrome (CIS) → relapsing-remitting MS (RRMS) → secondary progressive MS {SPMS}) and correlate with disability (Expanded Disability Status Scale {EDSS}, Multiple Sclerosis Functional Composite {MSFC})	Gray matter fraction (GMF), brain parenchymal fraction (BPF), white matter fraction (WMF), lesion volumes, MTR.	Disease-modifying drugs (DMTs) like interferon-β and glatiramer acetate have limited efficacy in secondary progressive multiple sclerosis.	Lack of MRI correlates for GM atrophy in SPMS; unclear mechanisms of GM pathology.	Gray matter (GM) atrophy is clinically relevant; underscores need for GM-focused measures in trials.	Need for advanced MRI techniques to study gray matter (GM) pathology, longitudinal studies across disease stages.
Williams et al. (2022) [31]	Randomized controlled trial (MS-STAT) with simvastatin vs. placebo.	Secondary progressive multiple sclerosis (SPMS)	Neuroaxonal injury reflected by neurofilament light chain (NfL) is linked to neuroinflammation. Simvastatin's neuroprotective effects may act independently of neuroaxonal injury pathways.	Serum NfL (neurofilament light chain), serum NfH (neurofilament heavy chain)	Higher NfL is associated with greater whole-brain atrophy, T2 lesion volume, and physical disability in SPMS. No significant associations found for NfH.	NFL and NFH were measured as potential biomarkers. NFL correlated with MRI measures of inflammation (T2 lesions) and neurodegeneration (brain atrophy). NfH showed limited utility as a biomarker in this context.	Simvastatin (80 mg) showed no significant effect on NfL (neurofilament light chain)/NfH {neurofilament heavy chain} levels despite reducing brain atrophy. NFL may not capture neuroprotective effects of non-immunomodulatory therapies.	NFL's utility may be limited for non-immunomodulatory treatments. NfH assay sensitivity and stability issues. Need for biomarkers capturing non-inflammatory neuroprotection.	NfL may aid in monitoring disease severity and progression in SPMS (secondary progressive multiple sclerosis). Simvastatin's neuroprotective effects warrant further validation in phase 3 trials.	Replicate NfL analysis in phase 3 multiple sclerosis - simvastatin trial 2 (MS-STAT2 trial). Develop novel biomarkers for neuroprotection independent of neuroinflammation. Explore mechanisms of simvastatin's effects on brain atrophy.
Lin et al. (2016) [18]	Design: nationwide population-based cohort study (Taiwan). Participants: 1,074 SLE patients vs. 5,370 age-/sex-matched controls.	Systemic lupus erythematosus (SLE)	Cerebrovascular: antiphospholipid antibodies → hypercoagulation → stroke/cerebral microinfarction → cognitive decline. Autoantibodies: anti-N-methyl-D-aspartate (NMDA) receptors, antibodies → hippocampal/amygdala damage. Neuroanatomical: hippocampal/cortical atrophy linked to SLE duration/autoantibodies. Steroid use: glucocorticoid receptor effects on memory/cognition.	Antiphospholipid antibodies and anti-NMDA receptor antibodies	SLE patients had a 2.14 times higher dementia risk (adjusted HR) vs. controls. Higher prevalence of hypertension, hyperlipidemia, and stroke in the SLE cohort. Affective psychosis (e.g., depression) increased dementia risk (HR=3.99).	ICD-9-CM codes for SLE/dementia. No direct biomarker measurement (claims data only).	No specific therapies were studied. Implication: steroid-sparing strategies may reduce dementia risk.	Diagnostic accuracy: ICD codes may misclassify NPSLE as dementia. Confounders: lack of data on lifestyle, autoantibodies, or SLE severity. Overlap: NPSLE (neuropsychiatric systemic lupus erythematosus symptoms) may mimic dementia.	Public health: need for dementia prevention strategies in SLE patients. Clinical: monitor cognitive function in SLE, especially with cardiovascular comorbidities or steroid use.	Investigate pathogenic mechanisms (e.g., autoantibodies, neuroinflammation). Validate findings with biomarker data (e.g., cerebrospinal fluid {CSF} and anti-NMDA antibodies). Anti-N-methyl-D-aspartate (NMDA) receptor antibodies. Study steroid alternatives to mitigate cognitive decline.
Kim et al. (2023) [19]	Nationwide retrospective cohort study on SLE and Parkinson’s disease (PD) risk in Korea.	Systemic lupus erythematosus (SLE).	Immune-mediated neuroinflammation (microglial activation, oxidative stress), dopaminergic neuron loss linked to autoimmune dysfunction, anti-dopaminergic antibodies, and vascular damage in SLE-associated Parkinson’s disease (PD).	TNF-α, antiphospholipid antibodies, and dopaminergic receptor dysfunction.	SLE patients had 1.59x higher risk of PD vs. controls (adjusted HR). Earlier PD onset in SLE patients.	ICD-10 codes (G20 for PD, M32 for SLE), rare intractable diseases (RID) program registration.	NSAIDs (e.g., ibuprofen) may protect against PD via the peroxisome proliferator-activated receptor gamma pathway (PPARγ pathway). Symptomatic dopaminergic drugs.	Lack of data on SLE disease activity/severity. It is unclear if PD risk is due to active inflammation or degenerative damage. Limited generalizability (Korean population only).	Clinicians should monitor SLE patients for PD symptoms. Public health burden of comorbid SLE and Parkinson’s disease (PD).	Investigate SLE-specific mechanisms (e.g., anti-dopaminergic antibodies). Prospective studies with disease activity metrics, explore race/ethnicity differences in PD-systemic lupus erythematosus (SLE) association.
Kim et al. (2023) [27]	Design: nationwide longitudinal cohort study (South Korea). Participants: 4,068,560 adults (14,722 with bronchiectasis vs. 4,053,838 controls). Follow-up: median 9.3 years (2009-2020).	Non-cystic fibrosis bronchiectasis.	Systemic inflammation: TNF-α, IL-6 → neurodegeneration via β-amyloid oligomerization. Chronic hypoxia: neuroinflammation, oxidative stress, synaptic dysfunction. Vascular mechanisms: endothelial damage from exacerbations (female/low-income subgroups).	TNF-α: tumor necrosis factor-alpha, IL-6: interleukin-6.	Bronchiectasis patients had 1.6× higher dementia incidence (15.0 vs. 9.3/1,000 PY). Alzheimer’s disease: 7% increased risk (aHR: 1.07, 95% CI: 1.01-1.12). Vascular dementia: no overall risk increase, but higher in women (aHR: 1.20) and low-income groups (aHR: 1.47).	International Classification of Diseases (ICD-10) codes for bronchiectasis/dementia. No direct biomarker measurement (claims data only).	Prevention: symptom control, physical activity, and cognitive training. Screening: early detection of Alzheimer’s disease.	Diagnostic accuracy: reliance on ICD codes. Confounders: lack of data on hypoxia severity or inflammatory biomarkers. Generalizability: Korean population only.	Public health: dementia screening for bronchiectasis patients. Clinical: manage exacerbations to reduce systemic inflammation/hypoxia.	Investigate inflammatory pathways (e.g., tumor necrosis factor-alpha (TNF-α), IL-6) in bronchiectasis-related dementia. Explore hypoxia mitigation strategies. Validate findings in diverse populations.
Zhang et al. (2024) [30]	Randomized controlled trial study exploring the association between COPD and psychiatric disorders using Genome-Wide Association Studies (GWAS) data. Subgroup analyses based on smoking history.	Chronic obstructive pulmonary disease (COPD).	Oxidative stress from smoking; shared genetic pathways.	Not specified.	Ever-smokers with COPD: increased ADHD risk; reduced Alzheimer’s risk. No association in never-smokers.	Genetic variants (SNPs): single-nucleotide polymorphism.	Smoking cessation.	Heterogeneity in SNPs; limited generalizability to non-European populations.	Focus on preventing psychiatric disorders in chronic obstructive pulmonary disease (COPD) patients with a smoking history.	Larger genome-wide association studies (GWAS) to identify more SNPs; broader population studies.
Tzeng et al. (2018) [20]	Nationwide, population-based cohort study on fibromyalgia and dementia risk in Taiwan.	Fibromyalgia (FM).	Systemic/brain inflammation (elevated pro-inflammatory cytokines). Hippocampal atrophy, neural noise hypothesis for cognitive dysfunction.	Plasma/CSP cytokines (e.g., IL-8, monocyte chemoattractant protein-1 {MCP-1}) are inflammatory pain markers.	FM patients had 2.77x higher risk of dementia. Subtypes: Alzheimer’s dementia (HR=3.35), vascular dementia (HR=3.14).	ICD-9-CM codes (FM: 411.1, 413, 414.0, 414.8-9; dementia: 290.x, 331.0) National Health Insurance Research Database (NHIRD) claims data.	Anti-inflammatory drugs (potential role, not studied) - symptomatic management of FM.	Lack of data on FM severity/duration. No pharmacological treatment analysis, protopathic bias (FM diagnosis after cognitive decline).	FM patients may need early cognitive screening. Higher dementia risk in low-income/urbanized FM patients.	Investigate FM-specific inflammatory pathways. Longitudinal studies with fibromyalgia (FM) severity metrics. Explore socioeconomic status (SES) and lifestyle confounders.
Abuhasira et al. (2019) [22]	Design: retrospective cohort study (Israel, 2000-2015). Participants: 2,606 PD patients (60 with fibromyalgia {FM}).	Fibromyalgia (FM) and Parkinson’s disease (PD).	Central sensitization: shared dopaminergic dysfunction (reduced dopamine metabolism in fibromyalgia (FM); dopaminergic neuron loss in Parkinson’s disease {PD}). Non-motor symptoms: overlapping pain, depression, and anxiety. Late-onset FM: may reflect PD prodromal stage or secondary central pain syndrome.	None directly measured (claims data).	Functional Limbic Interface Syndrome with Pain and Depression (FLISPAD) subgroup: 2.3% of PD patients had FM (88.3% female). Comorbidities: higher depression (43.3% vs. 20.9%), anxiety (23.3% vs. 7.2%), and dementia (35% vs. 22%). Medication use: increased analgesics (e.g., opioids, serotonin-norepinephrine reuptake inhibitors (SNRIs), and anti-PD drugs in FLISPAD patients.	International Classification of Diseases, 9th revision (ICD-9) codes for fibromyalgia (FM) and Parkinson’s disease (PD). Medication purchase records (e.g., APDs: antipsychotic drugs, SSRIs: selective serotonin reuptake inhibitors, and SNRIs: serotonin-norepinephrine reuptake inhibitors).	Pain management: opioids, SNRIs, SSRIs. PD treatment: more frequent antipsychotic drug (APD) switches in FLISPAD patients. Holistic care: address mental health comorbidities.	Diagnostic accuracy: FM diagnosed clinically (no biomarkers). Confounders: lack of data on pain severity or inflammatory pathways. Generalizability: Israeli population only.	Clinical: screen PD patients for FM-like symptoms, especially women with mental health comorbidities. Public health: optimize pain management strategies for Functional Limbic Interface Syndrome with Pain and Depression (FLISPAD).	Investigate dopaminergic pathways in Functional Limbic Interface Syndrome with Pain and Depression (FLISPAD). Validate fibromyalgia-Parkinson’s disease (FM-PD) overlap with biomarkers (e.g., CSF dopamine). Explore non-opioid therapies for centralized pain.
Letenneur et al. (2008) [21]	Population-based cohort study on HSV seropositivity and Alzheimer’s disease (AD) risk in France.	Herpes simplex virus (HSV) infection.	Herpes simplex virus (HSV) reactivation (IgM+) may cause chronic brain inflammation. Hippocampal damage (similar to HSV encephalitis), amyloid aggregation triggered by Herpes simplex virus (HSV) glycoprotein B homology.	Anti-HSV IgM (reactivation marker), anti-HSV IgG (past infection).	IgM+ subjects had 2.55x higher Alzheimer’s disease risk (HR). No association with IgG+ status. No interaction with APOE4 (apolipoprotein E epsilon 4 allele).	Serum IgM/IgG antibodies and Mini-Mental State Examination (MMSE) for cognitive decline.	Antiviral therapy (potential preventive strategy), HSV vaccination (hypothetical).	Single-time antibody measurement, no HSV subtype (HSV-1 vs. HSV-2) data, small sample for APOE4 (apolipoprotein E epsilon 4 allele) subgroup.	Herpes simplex virus (HSV) screening may identify high-risk elderly public health focus on viral infection control.	Longitudinal Herpes simplex virus (HSV) monitoring, HSV-1 vs. HSV-2 specificity, clinical trials for antivirals in AD prevention.
Torniainen-Holm et al. (2018) [23]	Longitudinal study (11-year follow-up) sample: Finnish adults (n=7112, aged 30+ years), representative of general population.	Cytomegalovirus (CMV) and Epstein-Barr virus (EBV) latent infections.	Hypothesized role of viral-induced inflammation in cognitive decline (not confirmed).	Cytomegalovirus (CMV) and Epstein-Barr virus (EBV) IgG antibody levels.	No significant association found between cytomegalovirus (CMV)/Epstein-Barr virus (EBV) and cognitive decline or dementia.	Seropositivity and serointensity of cytomegalovirus (CMV) and Epstein-Barr virus (EBV).	Not discussed.	Small effect sizes, lack of seroconversion data. Limited cognitive test battery.	High seroprevalence of cytomegalovirus (CMV) (84%) and Epstein-Barr Virus (EBV) (98%) in Finnish adults.	Need for studies on subpopulations (e.g., immunocompromised) or genetic susceptibility.

Challenges and Gaps

The review found limited generalizability due to homogeneous populations [13,15] and misinterpretation due to claims data and the lack of directly quantifiable biomarkers in SLE [18,20]. Cross-sectional designs hinder causal inference, and longitudinal studies have short follow-ups or no confounder information [21,24,25,32]. Many questions remain, including the role of neuroinflammation in T2D [24], differences in viral infection findings [23], and unexplored therapeutic opportunities, such as the ineffectiveness of MS therapies in progressive forms [28] or the potential of anti-inflammatory medicine in treating fibromyalgia and SLE [20]. Future researchers must address questions.

Clinical and Public Health Implications

In clinical and public health settings, chronic inflammatory disorders' effects on neurodegeneration demand unique interventions. RA's proactive therapy reduces Alzheimer's risk but not PD; however, lysosomal pathway protection is unknown [11,12]. Obesity in midlife predisposes one to dementia by 74%; thus, weight management is important, yet obesity in late life reduces dementia risk, showing that age matters [14]. To prevent cognitive decline in T2D, glycemic management (HbA1c <7%) is crucial [16,17]. Patients with SLE and FM should be examined early due to significant dementia risks [18,20]. Public health should prioritize condition-specific surveillance, lifestyle treatments, and biomarker integration. T2D and MS biomarkers like GFAP and NfL can give actionable information, but SLE and FM diagnosis accuracy, mainly based on claims data, is still lacking [31]. Addressing these issues and examining diverse populations would enhance clinical and preventative care [11,27].

Future Research Recommendations

Mechanistic, longitudinal, and multi-population research are needed to study chronic inflammation and neurodegeneration. Research on RA highlights the need to investigate the protective impact of lysosomal dysfunction in PD utilizing larger and diverse cohorts [11,12]. Researchers recommend investigating adipocytokines, neuroinflammation, and biomarkers such as GFAP and NfL in metabolism-related disorders like T2D and obesity to clarify their roles independent of amyloid and tau pathology [24]. SLE and FM are autoimmune disorders that need confirmation of inflammatory markers (anti-NMDA antibodies, IL-8) and study of central sensitization processes [18,20]. Clinical trials could test antivirals for Alzheimer's prevention in situations of viral infections like HSV, while neuroimaging investigations for vascular disorders should analyze overlapping pathologies [25,32]. The homogenous research group and retrospective or claims data restrict the generalizability of a set of studies [15,19]. Future research should prioritize multi-omics (genomics, proteomics) and focused therapies (lysosomal modulation in RA or glycemic control in T2D), although condition-specific processes must be addressed [16,17]. Additionally, viral and autoimmune-induced neurotoxicity studies should involve poorly represented groups and more rigorous longitudinal designs to evaluate causation and therapeutic potential.

## Conclusions

Neurodegeneration due to chronic inflammation is linked to shared mechanisms, such as systemic inflammation, microglial activation, and metabolic dysfunction. Although RA is associated with PD protection through lysosomal pathways, it does cause an AD risk. However, obesity and T2D have a consistently negative effect on cognitive deterioration. Dementia is further aggravated by autoimmune diseases (e.g., SLE) and central sensitization disorders (e.g., FM), which affect the body through neuroinflammation and cause vascular damage. Existing treatment therapies, such as immunomodulation and glycemic control, are promising and lack precision through biomarker and mechanistic gaps. These restrictions can be discussed and avoided with longitudinal studies and more specific interventions. Finally, the multidisciplinary approach to inflammatory disease detection and management could present key management interventions to postpone or even avert neurodegenerative diseases.
